# Identification of Hub Genes of Lung Adenocarcinoma Based on Weighted Gene Co-Expression Network in Chinese Population

**DOI:** 10.3389/pore.2022.1610455

**Published:** 2022-08-10

**Authors:** Yuning Xie, Hongjiao Wu, Wenqian Hu, Hongmei Zhang, Ang Li, Zhi Zhang, Shuhua Ren, Xuemei Zhang

**Affiliations:** ^1^ School of Public Health, North China University of Science and Technology, Tangshan, China; ^2^ Affiliated Tangshan Gongren Hospital, North China University of Science and Technology, Tangshan, China; ^3^ College of Life Sciences, North China University of Science and Technology, Tangshan, China

**Keywords:** next-generation sequencing, survival analysis, lung adenocarcinoma, WGCNA, PPI network

## Abstract

**Purpose:** Lung adenocarcinoma is one of the most common malignancies. Though some historic breakthroughs have been made in lung adenocarcinoma, its molecular mechanisms of development remain elusive. The aim of this study was to identify the potential genes associated with the lung adenocarcinoma progression and to provide new ideas for the prognosis evaluation of lung adenocarcinoma.

**Methods:** The transcriptional profiles of ten pairs of snap-frozen tumor and adjacent normal lung tissues were obtained by performing RNA-seq. Weighted gene co-expression network analysis (WGCNA) was used to construct free-scale gene co-expression networks in order to explore the associations of gene sets with the clinical features and to investigate the functional enrichment analysis of co-expression genes. Gene Ontology (GO), Kyoto Encyclopedia of Genes and Genomes (KEGG) pathway, and Gene Set Enrichment Analysis (GSEA) analyses were performed using clusterProfiler. The protein-protein network (PPI) was established using the Search Tool for the Retrieval of Interacting Genes/Proteins (STRING) and hub genes were identified using Cytohubba in Cytoscape. Transcription factor enrichment analysis was performed by the RcisTarget program in R language.

**Results:** Based on RNA-seq data, 1,545 differentially expressed genes (DEGs) were found. Eight co-expression modules were identified among these DEGs. The blue module exhibited a strong correlation with LUAD, in which *ADCY4*, *RXFP1*, *AVPR2*, *CALCRL*, *ADRB1*, *RAMP3*, *RAMP2* and *VIPR1* were hub genes. A low expression level of *RXFP1*, *AVPR*2, *ADRB1* and *VIPR1* was detrimental to the survival of LUAD patients. Genes in the blue module enriched in 86 Gene Ontology terms and five KEGG pathways. We also found that transcription factors *EGR3* and *EXOSC3* were related to the biological function of the blue module. Overall, this study brings a new perspective to the understanding of LUAD and provides possible molecular biomarkers for prognosis evaluation of LUAD.

## Introduction

Worldwide, lung cancer remains the major public health problem, which is the second most common cancer and the first cause of cancer-related death [1]. Non-small cell lung cancer (NSCLC) is the major type of lung cancer, which accounts for around 85% of all lung cancer cases. Tobacco smoking, diet and alcohol, ionizing radiation are all risk factors of NSCLC. Genetic factors, such as genetic polymorphisms and high-penetrance genes, also have great effect on the occurrence, development, and prognosis of NSCLC [2].

NSCLC is a multi-factorial disease. There is no clear conclusion regarding its etiology in the medical field. The main subtypes of NSCLC are squamous cell carcinoma (SCC), adenocarcinoma (ADC), and large-cell carcinoma (LCC) [3, 4]. Nowadays, the therapeutic choice of NSCLC largely based on the histopathological features, but the survival rates of NSCLC patients remain unsatisfactory. Studies showed that therapeutic progress for NSCLC was also attributed to specific genomic aberrations, which might serve as the molecular target. It is important to identify cancer subtypes based on common molecular features, which may benefit for patients with NSCLC.

With the advanced progress of high-throughput sequencing techniques, there is vast amount of data produced, which brings a big challenge for researchers to discover the pathways and key genes related to certain diseases. For RNA-seq data, researchers usually used conducted functional annotation, including GO, KEGG enrichment and Gene Set Enrichment analysis (GSEA) analyses [5]. This will lead to a profound understanding of the pathways by which cancer commonly evolves. However, there is still one major drawback due to the lack of interaction analysis between genes. Most studies used differential expression patterns as a screening standard. The inherent characteristics of expression profile data implies the genes with the greatest changes in expression levels are not necessarily the genes that responsible for tumor progression. The complex hierarchical relationships within the biological regulatory network remain to be fully explored.

Recently, the newly effective algorithms have been developed to better interpret big data from RNA sequencing. Weighted gene co-expression network analysis (WGCNA) is a systems biology method for describing the correlation patterns among genes based on the similarities of gene expression profiles [6]. Using expression data from cancer and adjacent normal tissue, WGCNA has been widely applied in detecting stage-specific gene co-expression modules and the hub genes within each module, which has the potential of pointing towards biologically and clinically relevant disease mechanisms [7, 8]. Constructure of protein-protein interaction (PPI) network is essential to understand the physiology of cells in normal and disease status within different modules. WGCNA analyses integrated with PPI network analysis will better identify and retrieve the signatures of hub genes. In addition, WGCNA has been successfully utilized to investigate the relationship between gene sets and clinical traits for identification of candidate cancer biomarkers for various cancers, including breast cancer [9-11], colon adenocarcinoma [12, 13], esophageal carcinoma [14, 15] and stomach adenocarcinoma [16-17].

In this study, gene expression matrix was constructed based on high-throughput RNA sequencing and differentially expressed genes (DEGs) were analyzed. The DEGs identified were subjected to GO and KEGG enrichment analysis for each group, which were further validated using GSEA analysis. WGCNA was constructed to identify the key modules in lung adenocarcinoma. To further reveal the role of genes in the key module and identify hub genes, KEGG pathways, GO enrichment, PPI network and transcription factor enrichment were conducted. The key genes in key module might serve as potential biomarkers for predicting the progression and the prognosis of lung adenocarcinoma.

## Materials and Methods

### Clinical Specimens

Ten pairs of snap-frozen lung cancer tissues and adjacent normal tissues were collected in Affiliated Tangshan Gongren Hospital of North China University of Science and Technology (Tangshan, China). All specimens were obtained at the time of surgery and confirmed by pathological examination. All patients were genetically unrelated Han Chinese, none of which had received preoperative chemotherapy, radiotherapy, or targeted therapy when recruited. This study is approved by the institutional review board from the Human Ethics Review Committee of North China University of Science and Technology (2022027) and Technology and informed consent was obtained from each patient.

### RNA-Sequencing and Data Pre-Processing

Total RNAs were extracted with Trizol reagent (Invitrogen, United States) following by the manufacturer’s protocol. RNA quality and integrity were analyzed using NanoPhotometer spectrophotometer (IMPLEN, Germany) and Agilent 2100 Bioanalyzer (Agilent Technologies, United States). To build RNA-seq libraries, ribosomal RNA (rRNA) was removed from total RNA to obtain all mRNA and lncRNA which were then randomly interrupted. The RNA-seq libraries were constructed using Illumina Truseq™ RNA sample prep Kit and were sequenced on the NovaSeq 6000 system. Before bioinformatics analysis, FastQC (http://www.bioinformatics.babraham.ac.uk/projects/fastqc/) was used to assess the quality of raw data and pre-process the raw data to obtain high quality clean reads data. Cleaned reads were then mapped to the human reference genome GRCh38/hg38 using spliced-reads aligner HISAT2 [18] and StringTie [19] to obtain raw read counts and transcripts per million (TPM).

### Gene Expression Analyses

Raw read counts data were used for gene expression analyses. Genes with low counts might represent a bias of sequencing and contribute less to further analysis, so we excluded genes with zero expression values. After data filtering**,** a total of 20,431 genes remained. DESeq2 normalized fold-change was used to analyze the differential gene between lung cancer tissues and adjacent normal tissues using the Bioconductor package DESeq2 [20]. Differentially expressed genes were defined as *p*-value < 0.05 and absolute log_2_ (fold change) (|log_2_FC|) ≥ 1. The results were represented by a volcano map and heatmap. The raw sequence data reported in this paper have been deposited in the Genome Sequence Archive in National Genomics Data Center (GSA-Human: HRA002426) that are publicly accessible at https://ngdc.cncb.ac.cn/gsa-human, while raw counts were provided in Supplementary Table S18.

### Pathway and Function Enrichment Analyses

To identify the biological function of differentially expressed genes (DEGs) in the development of lung cancer, functional enrichment Gene Ontology (GO, http://geneontology.org/) and Kyoto Encyclopedia of Genes and Genomes (KEGG, http://www.kegg.jp/) pathway analyses were performed using the Bioconductor package clusterProfiler [21]. Gene Ontology terms were divided into three separate subgroups: molecular functions (MFs), cellular components (CCs) and biological processes (BPs). Enriched GO terms and KEGG pathways were identified according to the cut-off criterion of *p*-values < 0.05. In addition, Gene Set Enrichment Analysis (GSEA) was performed for the complete expression profile using clusterProfiler. The GSEA results could be used as further validation of GO and KEGG enrichment results based on DEGs. To elucidate the biological processes of proteins in key module and their role in signaling transduction, ClueGO plugin of Cytoscape was used to perform KEGG pathway analysis and GO functional annotation [22]. *P*-value < 0.05 corrected by Bonferroni method were considered as significance.

### Weighted Gene Co-Expression Network and Their Key Modules

Weight gene co-expression network analysis (WGCNA) could construct a scale-free network based on gene expression profiles. In this study, weighted gene co-expression network was constructed using the Bioconductor package WGCNA [6]. Firstly, transcript per million (TPM) value expression matrix of EDGs was loaded into R. Based on TPM value, a hierarchical clustering analysis was performed. Secondly, the optimal soft threshold β was screened based on Pearson’s correlation coefficient and to enhance strong connections and disregard weak correlations between genes in the adjacency matrix. Then, the adjacency matrix was converted into a TOM to describe the association strength between the genes, and DynamicTreeCut algorithm was determined to construct a scale-free network. TPM expression matrix was loaded into the WGCNA package to get key modules and corresponding Eigengenes (MEs), which representing the overall level of gene expression in individual modules. After calculating the dissimilarity of the module eigengenes and hierarchically clustered the modules, we merged correlated modules (*r* ≥ 0.75) as similar modules. By setting the minimum number of genes to 50, dissimilarity of the module eigengenes (MEs) was identified by moduleEigengenes function of WGCNA to assess the effect of these modules on clinical characteristics. The analysis code is accessible from GitHub code repository: https://github.com/xyn1115/code_for_WGCNA.

### Protein-Protein Interaction Network and the Identification of Hub Genes

Genes within the same module might play similar roles and have high connectivity. The protein-protein network (PPI) of genes the key module was established using the Search Tool for the Retrieval of Interacting Genes/Proteins (STRING, https://string-db.org/) [23]. In order to identify hub genes in the PPI, algorithm Maximal Clique Centrality (MCC) was used by the Cytohubba [24] plugin based on Cytoscape.

### Transcription Factor Binding Motifs Enrichment Analysis

Transcription factor binding motifs (TFBMs) enrichment analysis was performed using the Bioconductor package RcisTarget [25]. Firstly, annotation to motifs of transcription factors (TFs) in Homo sapiens were downloaded (https://resources.aertslab.org/). Secondly, RcisTarget selected DNA motifs which were significantly over-represented in the surroundings of the transcription start site (TSS) of the candidate genes. Thirdly, the motifs which were annotated to TFs and had high normalized enrichment score (NES) were retained. Finally, for each motif and gene-set, genes which were ranked above the leading edge were predicted as the candidate target genes.

### Survival Analysis

To see whether these hub genes and transcription factors (TFs) were related to prognostic significance, survival analysis was performed using TCGAbiolinks in R [26]. Gene expression data and related clinical information of LUAD patients were obtained from the TCGA repository (https://cancergenome.nih.gov/). *P* value less than 0.05 was considered statistically significant. Survival curves were estimated with the Kaplan-Meier method and log-rank test. KMplot (http://kmplot.com/analysis), a web-based survival analysis tool which data was derived from Gene Expression Omnibus (GEO) dataset, was utilized as an independent validation dataset for prognosis analysis.

## Results

### Expression Profiles in Lung Adenocarcinoma

**FIGURE 1:**
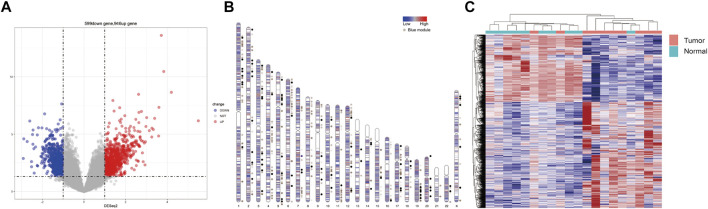
Differences in gene expression profile between lung adenocarcinoma and adjacent normal tissues. **(A)** Volcano plots showing differential expression of genes between the two groups. The red and blue points represent the differentially expressed genes; **(B)** The distribution of differentially expressed genes in human chromosomes. Dots indicate location of genes in blue module; **(C)** Hierarchical clustering analysis of all the genes.

After analyzing the differential expression of genes between lung adenocarcinoma tissues and adjacent normal tissues, 946 up-regulated and 599 down-regulated genes were identified in lung cancer tissues. The volcano plot presented DEGs between lung adenocarcinoma tissues and adjacent normal tissues (Figure 1A). The distribution of DEGs on human chromosomes was depicted in (Figure 1B). The hierarchical clustering results suggested that gene expression patterns were distinguishable between lung adenocarcinoma and control groups (Figure 1C).

### Gene Ontology and Kyoto Encyclopedia of Genes and Genomes Pathway Enrichment Analyses

**FIGURE 2:**
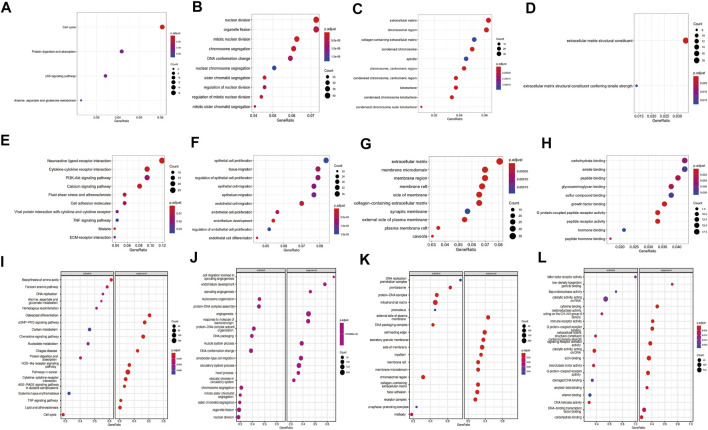
Enrichment analysis of differentially expressed genes (DEGs) and gene set enrichment analysis (GSEA) analysis of the complete expression profile. **(A)** KEGG pathways analysis for upregulated genes; GO analyses for upregulated genes including **(B)** biological process (BP), **(C)** cellular component (CC) and **(D)** molecular function (MF); **(E)** KEGG pathways are for downregulated genes; GO analyses for downregulated genes including **(F)** BP terms, **(G)** CC terms and **(H)** MF terms. **(I)** The enriched KEGG pathways by GSEA analysis, **(J)** BP terms **(K)** CC terms and **(L)** MF terms by GSEA analyses. Red dots indicate smaller p.adjust than blue dots. The size of the dots indicates the number of genes enriched in each analysis.

GO terms enrichment and KEGG pathway analyses of the DEGs were carried out to predict potential function of these DEGs in lung adenocarcinoma. For up-regulated genes, cell cycle, protein digestion and absorption, p53 signaling pathway and alanine, aspartate and glutamate metabolism pathway were enriched by KEGG analysis (Figure 2A, Supplementary Table S1). GO analysis revealed that up-regulated genes involved in the process of nuclear division, organelle fission and mitotic nuclear division in the biological process (BP) category; extracellular matrix, chromosomal region and collagen-containing extracellular matrix in cellular component (CC) category; extracellular matrix structural constituent and extracellular matrix structural constituent conferring tensile strength in molecular function (MF) (Figures 2B–D, Supplementary Tables S2–S4). For down-regulated genes, neuroactive ligand-receptor interaction, Malaria, cytokine-cytokine receptor interaction, calcium signaling pathway and fluid shear stress and atherosclerosis pathways were enriched KEGG analyses (Figure 2E, Supplementary Table S5). For down-regulated genes, the top enriched were associated with epithelial cell proliferation, tissue migration and regulation of epithelial cell proliferation in BP process; extracellular matrix, membrane microdomain and membrane region in CC process; carbohydrate binding, amide binding and peptide binding in MF process (Figures 2F–H, Supplementary Tables S6–S8).

GSEA analysis revealed significant activation or suppression of tumorigenesis-related genes. The most significantly activated pathways identified in this analysis include biosynthesis of amino acids, fanconi anemia pathway and DNA replication, while osteoclast differentiation, cGMP−PKG signaling pathway and chemokine signaling pathway were suppressed (Figure 2I). GSEA identified additional activated GO terms such as nucleosome organization (BP), DNA replication preinitiation complex (CC) and bitter taste receptor activity (MF). In contrast, suppressed GO terms include cell migration involved in sprouting angiogenesis (BP), external side of plasma membrane (CC) and low-density lipoprotein particle binding (MF) (Figures 2J–L). Complete GSEA results were provided in Supplementary Tables S9–S13.

### Weighted Co-Expression Network and Their Key Modules

**FIGURE 3:**
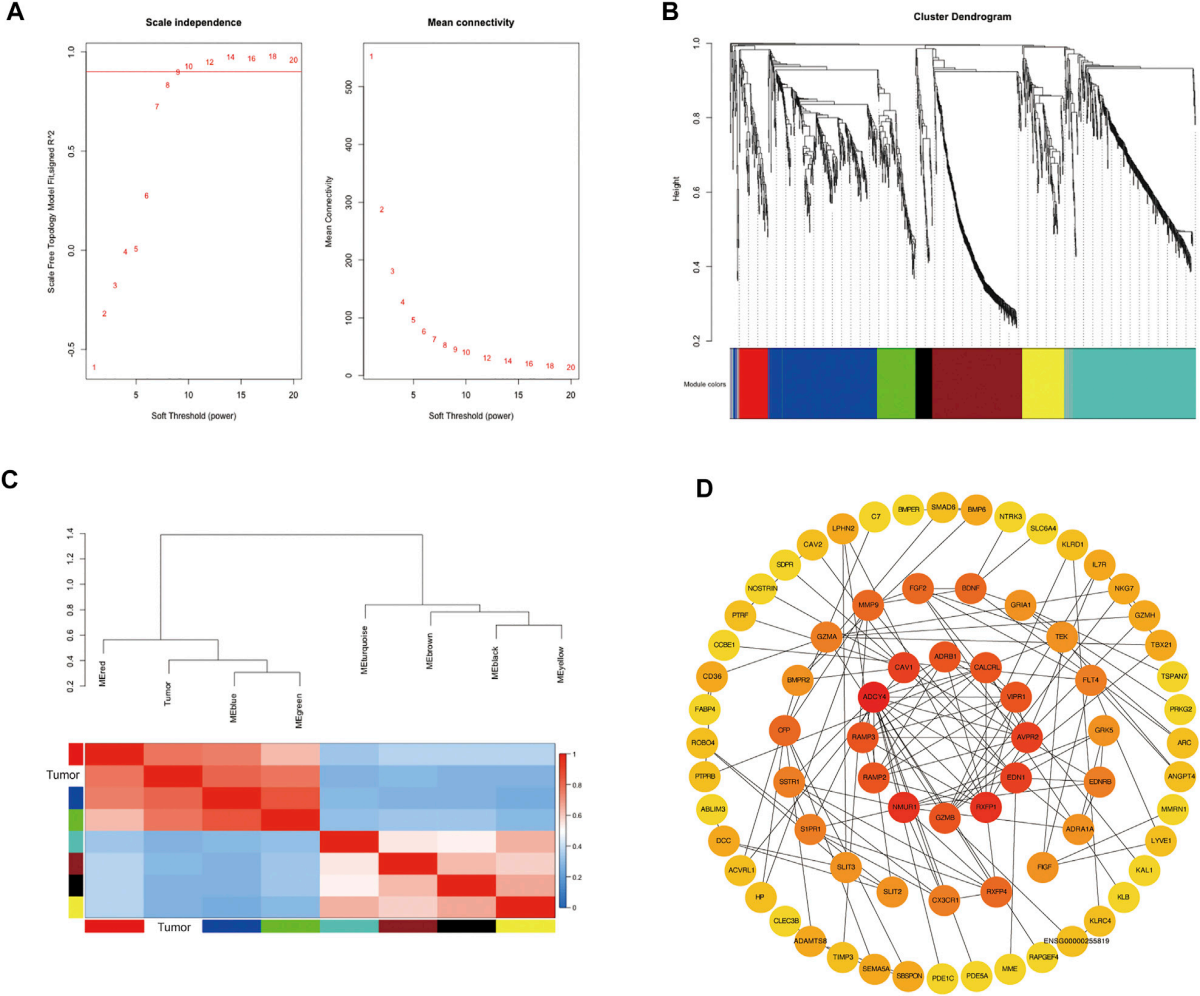
Identification of modules associated with the clinical status of lung adenocarcinoma in the WGCNA. **(A)** Analysis of the scale-free fit index and the mean connectivity for various soft-thresholding powers; **(B)** Hierarchical clustering dendrograms of identified co-expressed genes in modules in lung adenocarcinoma; **(C)** Heatmap plot of the adjacencies of modules, red represents high adjacency and blue represents low adjacency; **(D)** PPI analysis and identification of hub genes involved in the co-expression blue module using STRING database and cytoHubba plug-in in Cytoscape.

To further explore the co-expression patterns of the differential expression genes in lung adenocarcinoma, weighted co-expression network analysis (WGCNA) was performed. To ensure a scale-free network, we selected β value of 9 as the soft-thresholding power (Figure 3A). Eight co-expression modules were finally identified by the cluster dendrogram (Figure 3B). Different modules were represented by red, blue, green, turquoise, yellow, black, brown and grey and the number of genes in each module were 96, 366, 130, 418, 138, 55, 297 and 40, respectively. To evaluate the relationship between gene modules and lung adenocarcinoma, module eigengenes (MEs) which represented the gene expression profile of module, the correlation between module eigengenes (MEs) and lung adenocarcinoma were calculated to generate the eigengene adjacency heatmap (Figure 3C). Our result revealed that the blue module exhibited a strong correlation with lung cancer, indicating that blue module was the key module.

### Protein-Protein Interaction Network and Enrichment Analysis of the Differentially Expressed Genes in the Blue Module

**FIGURE 4:**
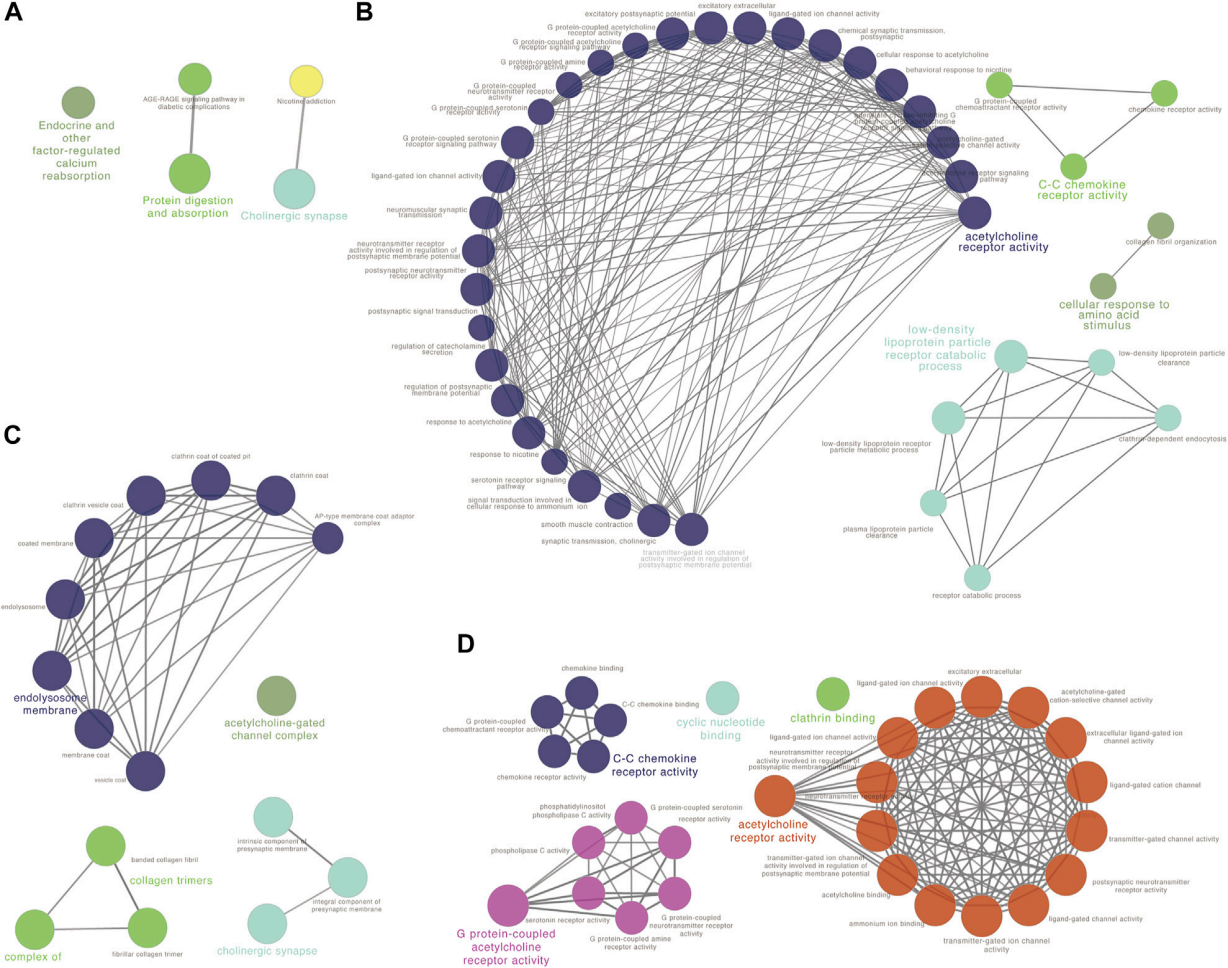
Function analysis for the blue module using ClueGO. **(A)** KEGG pathway analysis; **(B)** GO biological process (BP) analysis; **(C)** GO cellular component (CC) analysis and **(D)** GO molecular function (MF) analysis.

To reveal the function of the co-expressed genes in the blue module at the protein level, a protein-protein interactions network (PPI network) was constructed based on the STRING database (STRING, https://string-db.org/). The PPI network consisted of 74 nodes and 134 edges. Algorithm Maximal Clique Centrality (MCC) was performed to screen hub genes by cytoHubba plugin. We found that the top hub genes in the blue module included *ADCY4*, *RXFP1*, *AVPR2*, *CALCRL*, *ADRB1*, *RAMP3*, *RAMP2* and *VIPR1* (Figure 3D). To further clarify the biological functions of DEGs in the blue module, the co-expressed genes were annotated with KEGG pathway and GO terms using ClueGO plugin. Five KEGG pathways and GO terms for 40 biological processes, 16 cell components, and 30 molecular functions were identified (Figures 4A–D, Supplementary Tables S13–S16). Particularly, choline related function was significantly enriched in both KEGG and GO terms and C-C chemokine receptor activity also enriched in biological processes and molecular functions. These results implied that several of these terms in the blue module might work together to form a functional pathway contributing to lung adenocarcinoma.

### Survival Analysis of Hub Genes

**FIGURE 5:**
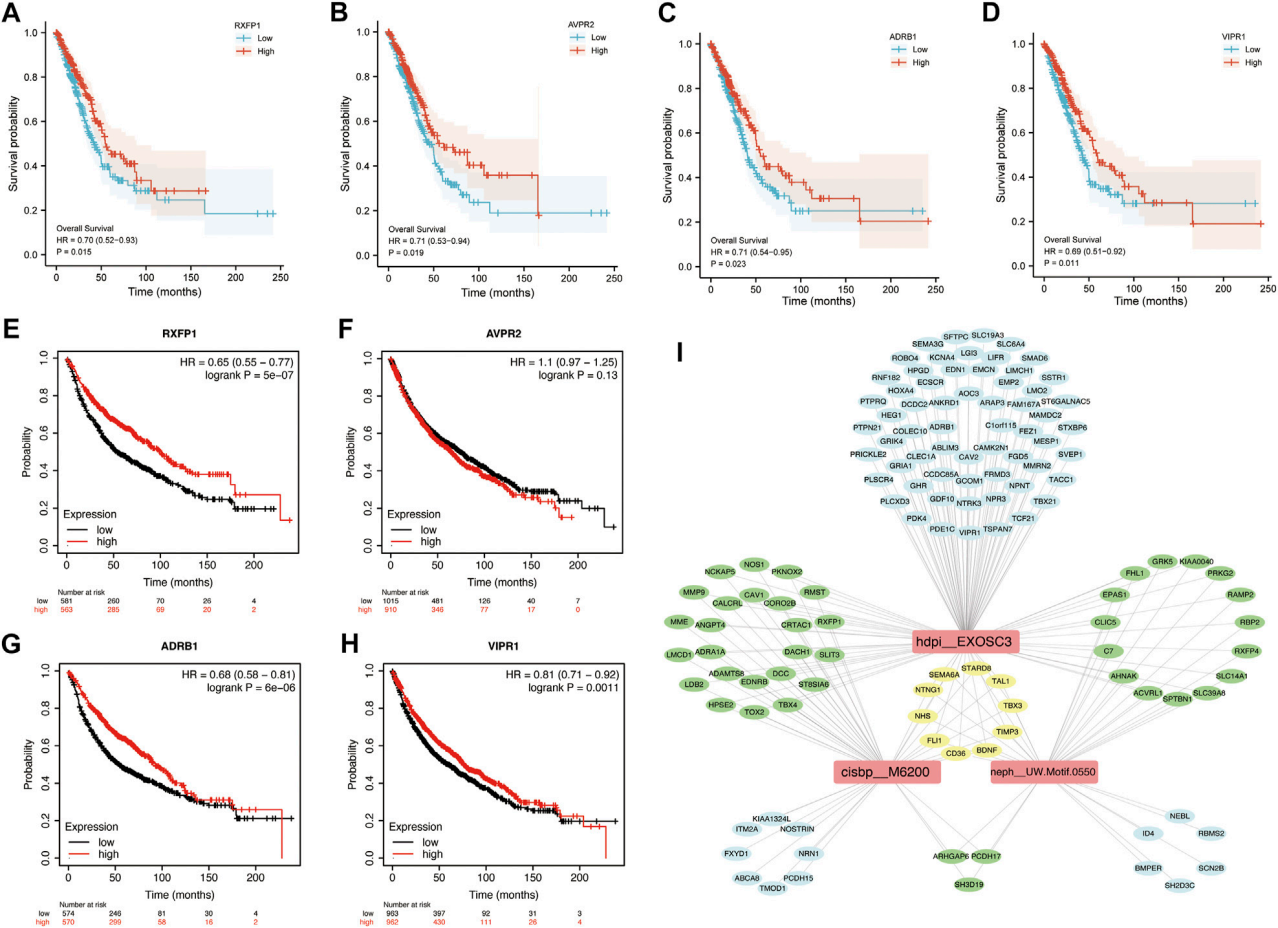
Kaplan-Meier Survival curves of hub genes and TFBMs analysis in blue module. K-M curves based on TCGA data. **(A)** *RXFP1*; **(B)** *AVPR2*; **(C)** *ADRB1*; **(D)** *VIPR1*. K-M curves based on KM plot database. **(E)** *RXFP1*; **(F)** *AVPR2*; **(G)** *ADRB1*; **(H)** *VIPR1*. Red curve represents patients with high expression of hub genes; **(I)** Transcription factor binding motifs (TFBMs) enrichment analysis.

To determine the potential value of hub genes in predicting the overall survival of LUAD patients, we analyzed the survival curves of patients based on TCGA data. Among the 8 hub genes in blue module, 4 genes were found to be statistically related to the overall survival rate (*p* < 0.05). LUAD patients with high expression of *RXFP1*, *AVPR2*, *ADRB1* or *VIPR1* had long overall survival rate. Kaplan-Meier survival analysis showed that the high expression of *RXFP1*, *AVPR2*, *ADRB1* and *VIPR1* were contributed to long overall survival time of LUAD patients with HR (95%CI) of 0.70 (0.52–0.93), 0.71 (0.53–0.94), 0.71 (0.54–0.95) and 0.69 (0.51–0.92), respectively (Figures 5A–D). In order to verify the reality of this finding, we performed survival analysis using validation dataset. KMplot database generated Kaplan–Meier curves based on public microarray datasets of lung cancer (GSE19188, GSE3141, GSE29013, GSE37745, GSE30219, GSE50081, GSE14814, GSE31908 and GSE4573). We demonstrated that the high expression of *RXFP1* (HR = 0.65, 95%CI = 0.55–0.77), *ADRB1* (HR = 0.68, 95%CI = 0.58–0.81) and *VIPR1* (HR = 0.81, 95%CI = 0.71–0.92) were significantly improved the overall survival rate. We didn’t find that *AVPR2* affect the prognosis of LUAD patients in validation dataset (*p* > 0.05) (Figures 5E–H).

### Transcription Factor Enrichment in the Blue Module

In order to reveal the influence of transcription factors on genes in blue module, transcription factor binding motifs (TFBMs) enrichment analysis was performed. As results, 27 TFBMs were enriched (Supplementary Table S17). The top 3 TF motifs were *cisbp_M6200*, *hdpi_EXOSC3* and *neph_UW.Motif.0550* (Figure 5I), which indicated that transcription factors *EGR3* and *EXOSC3* (*neph_UW.Motif.0550* had no direct annotation of TF) played a key role in the blue module. Interestingly, *NHS*, *SEMA6A*, *TBX3*, *FLI1*, *BDNF*, *NTNG1*, *TIMP3*, *STARD8*, *TAL1* and *CD36* were simultaneously regulated by three transcription factor binding motifs (TFBMs).

## Discussion

In this study, we identified 946 upregulated and 599 downregulated genes in lung adenocarcinoma. Calcium signaling pathway was enriched by KEGG analysis. Intracellular calcium (Ca^2+^), as an important second messengers, plays a variety of roles in basic cell physiology, including gene expression, cell cycle control, cell movement, autophagy and apoptosis [27]. The specific calcium signaling pathways have also been identified to be involved in the multidrug resistance [28].

GSEA analysis further revealed significant enrichment of *cGMP*−*PKG* signaling pathway. Piazza et al. revealed *cGMP*/*PKG* signaling activation could block cancer cell growth, *Wnt*/*β*-catenin transcription and tumor immunity [29]. Kong et al. found lncRNA *DARS*-*AS1* might activate *cGMP*-*PKG* pathway to accelerate tumor malignancy in cervical cancer [30]. Our GSEA analysis also indicated chemokine signaling pathway were suppressed. The chemokine *CXCL12*-*CXCR4*/*CXCR7* axis as a mechanism of tumor microenvironment and immune resistance in glioblastoma [31], bladder cancer [32], colorectal cancer [33] and gastrointestinal malignancies [34]. *CXCL13*/*CXCR5* signaling axis modulated cancer cell ability to grow, proliferate, invade, and metastasize [35]. Several studies showed the *CCL20*-*CCR6* axis was associated with several cancers, including hepatocellular carcinoma [36, 37], colorectal cancer [38, 39], breast cancer [40,41], and kidney cancer [42].

The main objective for this study was to utilize a global approach to construct a gene co-expression network and to predict clusters of candidate genes involved in the pathogenesis of lung adenocarcinoma. We hypothesized that tightly co-expressed gene modules, enriched in shared functional annotation, would provide the most effective predictions of candidate gene sets that might conduct basic biological functions. Modules changed significantly between lung adenocarcinoma tissues and normal tissues, but the blue module was the most significant. In geneset of blue module, we found that regulation of endothelial cell migration, membrane functions and G protein-coupled peptide receptor activity had been changed significantly. It was well known that migration and invasion were important features of tumors and always led to poor prognosis. The blue module might lie at the heart of lung adenocarcinoma. According to the PPI network analysis from the blue module, 8 high-degree hub genes were identified, which might play a critical role in the network. It was worth noting that the expression of *RXFP1*, *AVPR2*, *ADRB1* and *VIPR1* had significantly effect on the survival of patients with lung adenocarcinoma.

The Relaxin/relaxin family peptide receptor 1 (*RXFP1*) axis is an “old” pathway. Studies showed that *RXFP1* was associated with fibrotic diseases, such as lung fibrosis [43], kidney fibrosis [44] and cardiac fibrosis [45]. More recent studies suggested that Relaxin/*RXFP1*-mediated cancer growth and invasion in breast, thyroid and prostate cancers [46-51]**.** *RXFP1* also was involved in anti-apoptotic functions, angiogenesis and chemoresistance in cancer cells [52-56]. The arginine vasopressin type 2 receptor (*AVPR2*) agonist was able to impair tumor aggressiveness and distant spread in colorectal cancer [57]. *ADRB1* mutation was associated with lower tumor mutational burden and might serve as a potential clinical prognosis biomarker of breast cancer [58]. The vasoactive intestinal peptide receptor-1 (*VIPR1*) has prominent growth effects on a number of common neoplasms. The researchers found that the overexpression of *VIPR1* significantly inhibited the growth, migration, and invasion of in lung adenocarcinoma cells [59]. These studies implied that *RXFP1*, *AVPR2*, *ADRB1* and *VIPR1* might be involved in the development of cancer.

Transcription factors are involved in the development and prognosis of various cancers. *EGR3* loss was associated with prostate cancer progression and poor prognosis. In prostate cancer cells, *EGR3* blocked the EMT process and suppressed cell migration and invasion [60]. Li et al. found that Silencing of *miRNA-210* inhibited the progression of liver cancer *via* upregulating *EGR3* [61]. Chien et al. implied that *miR-23a* could directly bind to the 3'UTR of EGR3 to inhibit NSCLC cell mobility [62]. Ansari and his colleagues revealed that EXOSC3 was significantly upregulated in pancreatic cancer tissue using protein deep sequencing [63].

Despite traditional DEGs analysis has provided enormously relevant information; however, only WGCNA allowed for identifying correlation pattern among genes. In our study, we found strong correlation between the blue module and lung adenocarcinoma. In the blue module, *ADCY4*, *RXFP1*, *AVPR2*, *CALCRL*, *ADRB1*, *RAMP3*, *RAMP2* and *VIPR1* were identified as hub genes. Transcription factors *EGR3* and *EXOSC3* might play a regulatory role in gene expression in the blue module.

Taken together, after analyzing the expression data of LUAD, we identified 4 hub genes (*RXFP1*, *AVPR2*, *ADRB1*, and *VIPR1*) which might affect the prognosis of LUAD patients. However, further experiments are still needed to verify these hub genes and pathways.

## Data Availability

The datasets presented in this study can be found in online repositories. The names of the repository/repositories and accession number(s) can be found in the article/Supplementary Material.
